# Roles of claudin-2, ZO-1 and occludin in leaky HK-2 cells

**DOI:** 10.1371/journal.pone.0189221

**Published:** 2017-12-18

**Authors:** Sua Kim, Gheun-Ho Kim

**Affiliations:** 1 Institute of Biomedical Science, Hanyang University College of Medicine, Seoul, Korea; 2 Division of Nephrology, Department of Internal Medicine, Hanyang University College of Medicine, Seoul, Korea; Aix-Marseille University, FRANCE

## Abstract

**Background:**

Claudin-2, ZO-1, and occludin are major components of tight junctions (TJs) in the proximal tubule. However, their roles in maintaining paracellular permeability as leaky epithelia have yet to be defined.

**Methods:**

To investigate the contributory role of TJ proteins in the leaky proximal tubule, we xamined the effect of inhibiting claudin-2, occludin, and ZO-1 expression on transepithelial electrical resistance (TER) and paracellular permeability using the immortalized human proximal tubule epithelial cell line HK-2. For this, small-interfering RNAs (siRNAs) against claudin-2, occludin and ZO-1 were transfected into HK-2 cells. TER and transepithelial flux rates of dextrans (4 and 70 kDa) were determined after 24 h.

**Results:**

Transfection of siRNAs (25 nM) knocked down TJ protein expression. Control HK-2 monolayers achieved a steady-state TER of 6–8 Ω·cm^2^ when grown in 12-well Transwell filters, which are compatible with leaky epithelia. Knockdown of claudin-2 decreased in TER and increased occludin expression. Transfection with siRNA against either occludin or ZO-1 increased TER and decreased claudin-2 expression. TER was decreased by co-inhibition of claudin-2 and ZO-1 but increased by co-inhibition of claudin-2 and occludin. TER was suppressed when claudin-2, occludin, and ZO-1 were all inhibited. Dextran flux rate was increased by claudin-2, occludin, or ZO-1 siRNA transfection. Increased dextran flux was enhanced by co-transfection of claudin-2, ZO-1, and occludin siRNA.

**Conclusions:**

The depletion of claudin-2, occludin and ZO-1 in HK-2 cells had differential effects on TER and macromolecule flux. We demonstrated that integration of claudin-2, occludin and ZO-1 is necessary for maintaining the function of the proximal tubular epithelium.

## Introduction

Different renal tubular segments have specific epithelial characteristics and functions. For paracellular transport, the proximal tubule has leaky epithelia, whereas collecting ducts have more effective tight junctions (TJs). Paracellular permeability decreases from the proximal tubule to the collecting ducts because of a unique array of claudins expressed in each segment, with higher levels of occludin and ZO-1 in distal tubules [1]. Although claudin-2, occludin, and zonula occludens (ZO)-1 are major components of TJs in proximal tubules [2], their roles in maintaining “loose” epithelia have yet to be defined. Previous studies mainly focused on “tight” epithelia in collecting ducts.

TJs are multiprotein complexes of integral membrane proteins (claudins and occludin) and cytoplasmic scaffolding proteins (ZO-1, ZO-2 and ZO-3). Claudins are the backbone of TJ complexes involved in the selectivity of the paracellular conductance of ions. A number of claudins are expressed as barriers in the distal tubules and collecting ducts [3]. In contrast, claudin-2 may have a different function in loose epithelia because it is highly expressed in the proximal tubule [2].

Recent studies have shown that claudin-2 forms paracellular channels for small cations such as sodium and potassium and for water [4]. Occludin and ZO proteins interact to determine TJ structural organization and to create a classical barrier to the diffusion of solutes through the paracellular pathway [5, 6]. However, in the proximal tubule where high permeability is required for 70% of glomerular filtration reabsorption, the functions of occludin and ZO-1 remain unclear.

Traditionally, TJs have mainly been recognized as paracellular diffusion barriers. Recent studies established the involvement of TJ proteins in the formation of pores that serve as TJ pathways for the flux of small solutes and ions [7]. TJ proteins form a barrier-like structure through cis-interactions with other TJs in the same cell and trans-interactions with TJs in adjacent cells. Pore pathways are mainly involved in ionic and fluid transport and function under normal physiological conditions [8]. The pore functions of the TJ pathway are likely more important in the proximal tuble, in contrast with the barrier function in the distal nephron.

In this study, we used HK-2 cells as an immortalized proximal tubule epithelial cell line from normal adult human kidneys [9]. We investigated the pore and barrier functions of TJ proteins in leaky proximal tubule epithelial cells. For this, proximal tubule TJ proteins were selectively knocked down by small-interfering RNA (siRNA) transfection into HK-2 cells [10]. The inhibitory effects on endogenous expression of claudin-2, occludin and ZO-1 in HK-2 cells were examined by measuring transepithelial electrical resistance (TER) and dextran flux rates.

## Materials and methods

### Cell culture and siRNA transfection

HK-2 human renal proximal tubular epithelial cells (American Type Culture Collection, Manassas, VA) were cultured in Dulbecco’s modified Eagle’s and Ham’s F-12 medium (DMEM/F12, GIBCO) supplemented with 10% fetal bovine serum, 100 U/mL penicillin, and 100 mg/mL streptomycin. For depletion of TJ genes, siRNAs against claudin-2 (Cat No. 1032301 Duplex, Bioneer, Daejeon, Korea), ZO-1 (Cat No. 1151510 Duplex, Bioneer, Daejeon, Korea), and occludin (Cat No. L-187897-00-0005, Dharmacon, Lafayette, CO) were transfected into HK-2 cells using DharmaFect transfection reagents (Dharmacon, Lafayette, CO). Cells were grown to subconfluency at 37°C with 5% CO_2_ and treated with 10, 25, 50 or 100 nM of siRNA for 72 h.

### Immunoblot analysis

TJ protein levels were tested using semiquantitative immunoblotting. HK-2 cells were plated onto 100 mm-Petri dishes at 0.5 ~ 1 × 10^5^ cells/mL and cultured for siRNA transfection. Cells were harvested and lysed in lysis buffer (Santacruz, Heidelberg, Germany) containing protease inhibitor cocktail (Roche, Basel, Switzerland) for 30 min on ice. Cell lysates were centrifuged at 14,000 x *g* for 20 min at 4°C and protein concentrations measured using Bradford protein assay kits (Bio-Rad Laboratories, Hercules, CA). Equal amounts of protein were electrophoresed on SDS-polyacrylamide gels, transferred onto nitrocellulose membranes and blocked in 5% skim milk in PBST (80 mM Na_2_HPO_4_, 20 mM NaH_2_PO_4_, 100 mM NaCl, 0.1% Tween-20, pH 7.5) for 1 h. Membranes were probed overnight at 4°C with primary antibodies: rabbit polyclonal anti-occludin, rabbit polyclonal anti-ZO-1 or mouse monoclonal anti-claudin-2 (Zymed Labs, Jerusalem, Israel). Secondary antibodies were goat anti-rabbit or goat anti-mouse IgG conjugated to horseradish peroxidase (Jackson ImmunoResearch, West Grove, PA). Sites of antibody-antigen reaction were viewed using enhanced chemiluminescence (GenDEPOT, Barker, TX), and band densities were quantified by densitometry using a laser scanner and Quantity One Software (Basic version 4.6.9, Bio-Rad, Hercules, CA).

### Immunofluorescence microscopy of HK-2 cells

HK-2 cells were grown to confluence in transwell chambers (0.4-μm pore size, Transwell Permeable Supports, Cat No. 3460, Corning) for 3 days. On day 4, HK-2 cells were treated with vehicle or 10, 25, 50 or 100 nM siRNA for 72 h and fixed with 4% paraformaldehyde in PBS, pH 7.4 for 20 min at room temperature. After fixation, cells were washed twice in PBS and permeabilized with 0.3% Triton X-100 in PBS at room temperature for 15 min. Cells were washed and labeled with anti-claudin-2, anti-occludin or anti-ZO-1. After incubation, cells were washed in PBS and incubated with goat anti-rabbit IgG Alexa Fluor 488 secondary antibody (A11008, Molecular Probes) or donkey anti-mouse IgG Alexa Fluor 488 secondary antibody (A21202, Molecular Probes) for 2 h at room temperature. Immunolocalization used a laser scanning confocal microscope (Zeiss LSM 5 EXCITER, Jena, Germany) [11].

### Transepithelial electrical resistance

The intactness of paracellular pathways of HK-2 monolayers to small ions was monitored by measurement of TER using the EVOM apparatus (World Precision Instruments, Sarasota, FL). HK-2 cells that were seeded onto Transwell filters (diameter 12 mm, pore size 0.4 μM; Corning, NY) to measure TER after exposure to siRNA for 72 hours in apical chambers. TER was normalized to the area of the filter after removal of background resistance of a blank filter with medium only. TER was calculated as ohms × cm^2^ (Ω·cm^2^), after subtracting values for resistance of membrane support alone [12].

### Paracellular permeability

Effects of TJ siRNA transfection on HK-2 cell paracellular permeability were determined using Fluorescein isothiocyanate (FITC)-conjugated 4 and 70 kDa dextran (Sigma, Saint Louis, MO). Transwell filters of epithelial monolayers were transferred to 6-well culture dishes and FITC-dextran was added to apical compartments. After 24 h at 37°C, fluorescence was measured for aliquots of basolateral medium using a fluorimeter (Tecan Systems, San Jose, CA) [13].

### Statistics

Statistical analyses were performed with GraphPad Prism 5 (GraphPad Software, California, USA). Data were expressed as means ± standard deviation of experiments with at least three independent treatments per group. Differences between groups were analyzed by Student’s *t*-test for unpaired data. Statistical significance was defined as *P*<0.05.

## Results

### siRNA knockdown of claudin-2, occludin and ZO-1 in HK-2 cells

**Fig 1:**
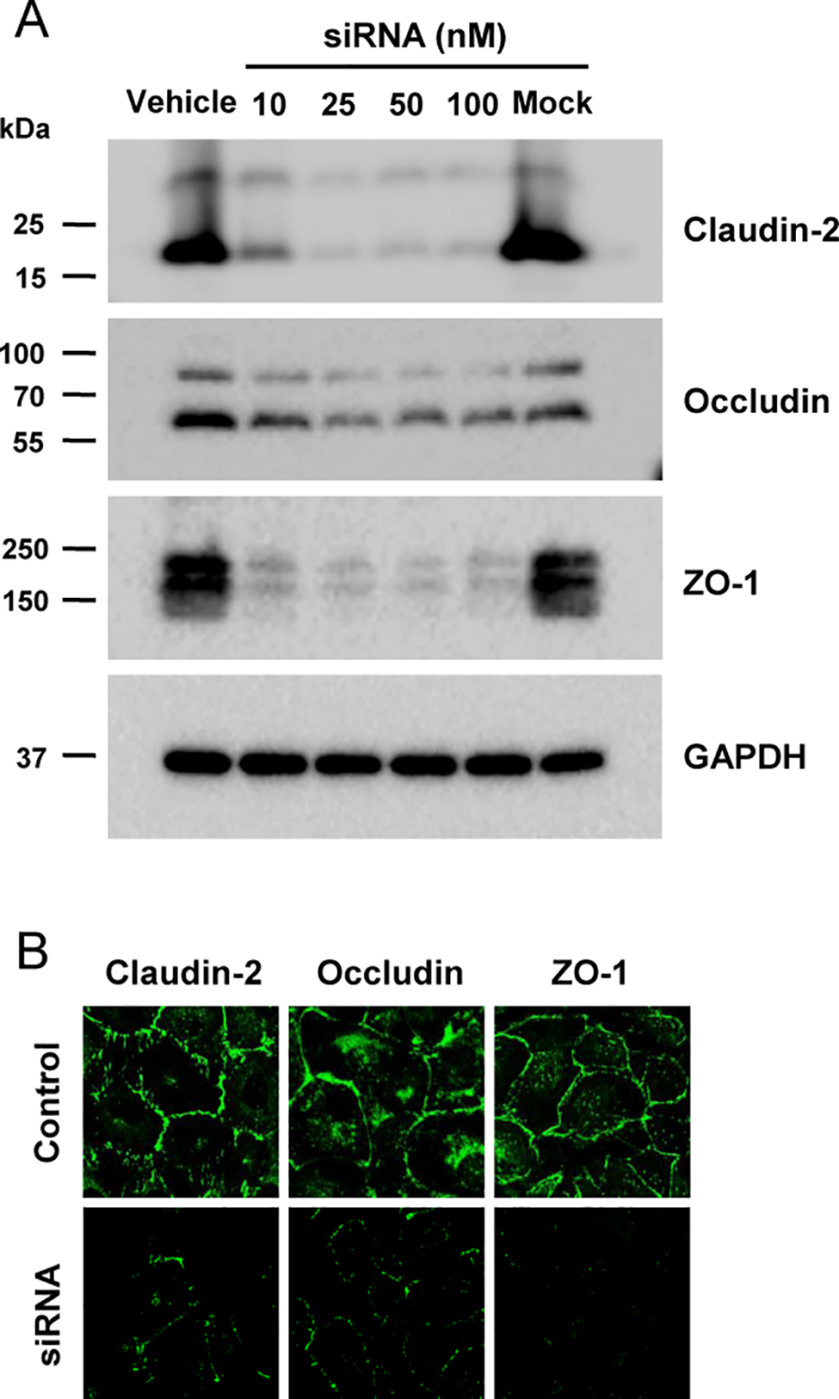
Results of transfection into HK-2 cells of siRNA against claudin-2, occludin or ZO-1. A: Immunoblots using cells treated with 10, 25, 50 of 100 nM of indicated siRNA for 72 h. Each lane contained protein from a different Transwell filter support of HK-2 cells. Immunoblots were treated with anti-claudin-2 (~22 kDa), anti-occludin (~65 kDa) or anti-ZO-1 (~210 kDa). B: Immunofluorescence microscopy of HK-2 monolayers with junctional localization of claudin-2, occludin and ZO-1 depleted by siRNA transfection. Magnification, x40.

siRNA-induced knockdown of TJ proteins in HK-2 cells are in Fig 1. Expression of claudin-2, occludin and ZO-1 was examined by immunoblotting after treatment with 10, 25, 50 or 100 nM siRNA. Transfection with siRNA against claudin-2, ZO-1, or occludin reduced the respective TJ protein (Fig 1A). At 25 nM siRNA, silencing efficiency was confirmed by immunofluorescence microscopy (Fig 1B).

**Fig 2:**
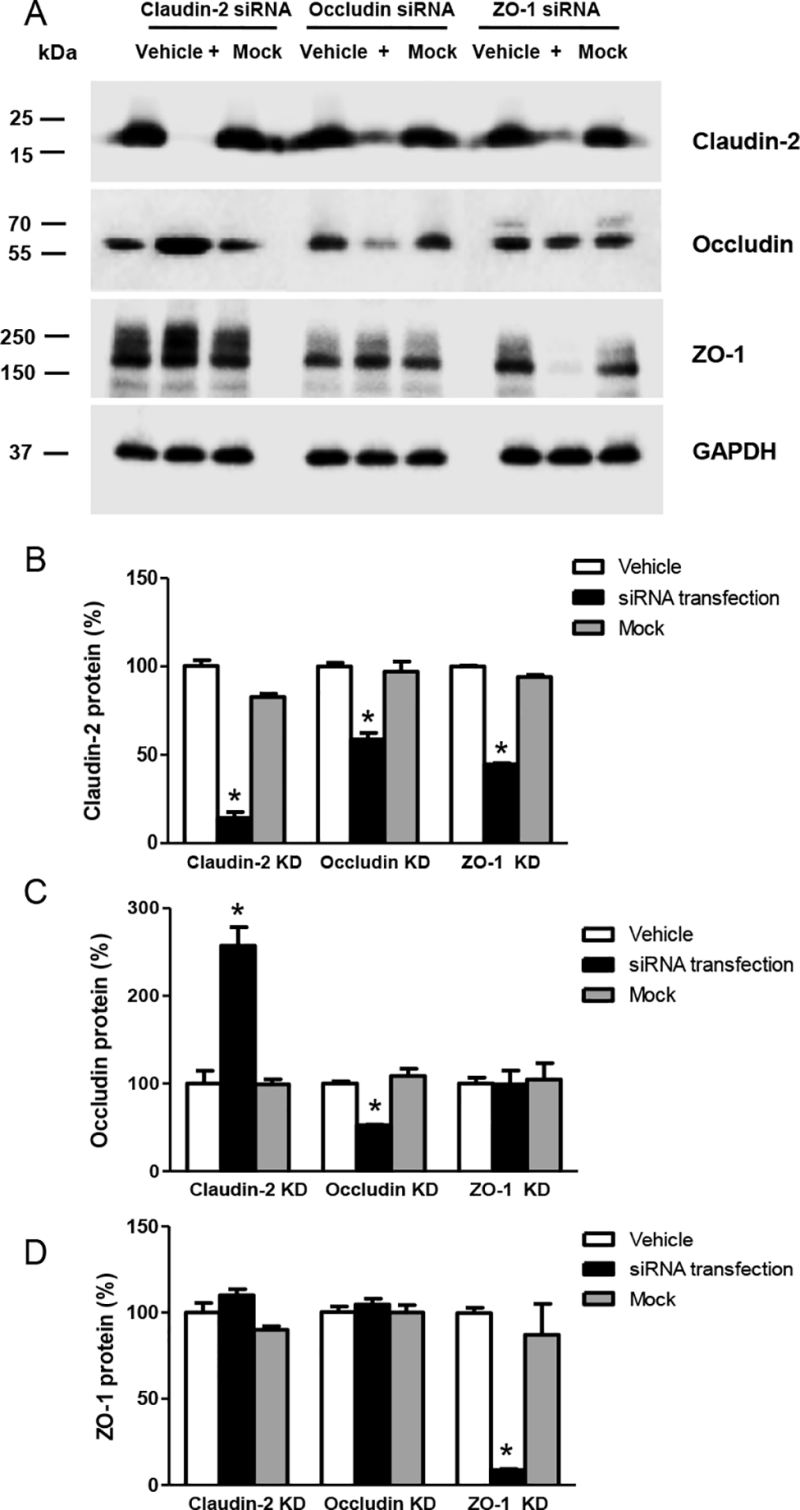
Effects of claudin-2, occludin and ZO-1 siRNA transfection on expression of other tight junction proteins in HK-2 cells. A: Immunoblots with antibodies to claudin-2, occludin, or ZO-1 using cells transfected with 25 nM indicated siRNAs for 72 h. GAPDH was the loading control. B: Densitometric analysis showing claudin-2, occludin, or ZO-1 siRNA significant decreased claudin-2 protein. C: Overexpression of occludin protein induced by claudin-2 siRNA transfection. D: Expression of ZO-1 protein was not affected in HK-2 cells by claudin-2 or occludin siRNA transfection. Quantification of data is % volume difference in protein expression, expressed as mean ± SD from 3 independent experiments. *P < 0.05 by Student’s *t*-test for unpaired data.

We found that siRNA-induced knockdown of claudin-2, occludin or ZO-1 accompanied alteration of the expression of other TJ proteins in HK-2 cells (Fig 2). Claudin-2 protein expression was significantly reduced by claudin-2 siRNA (14 ± 6%, *P* < 0.05) or transfection of siRNA against occludin (59 ± 6%, *P* < 0.05) or ZO-1 (45 ± 1%, *P* < 0.05). Occludin protein expression was significantly decreased by occludin siRNA (53 ± 1%, *P* < 0.05) and significantly elevated by siRNA transfection against claudin-2 (257 ± 37%, *P* < 0.05). ZO-1 expression was significantly suppressed by ZO-1 siRNA (8 ± 1%, *P* < 0.05), but not affected by either claudin-2 or occludin siRNA transfection.

### Effects of claudin-2, occludin and ZO-1 siRNA transfection on TER and dextran permeability

**Fig 3:**
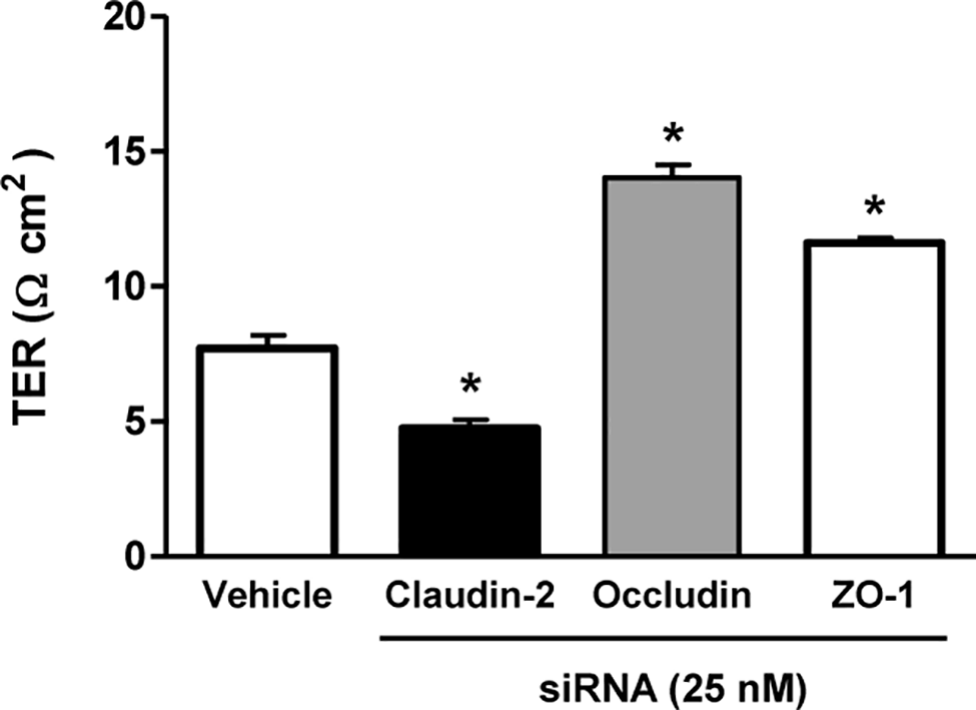
Effects of claudin-2, occludin or ZO-1 siRNA transfection on transepithelial electrical resistance (TER) in HK-2 cell monolayers. TER was significantly decreased by claudin-2 siRNA transfection, but significantly increased by siRNA transfection against occludin or ZO-1. Data are means ± SD. *P < 0.05 by Student’s *t*-test for unpaired data.

To examine the function of TJ proteins in maintenance of HK-2 cell monolayers, TER was compared after inhibition of claudin-2, ZO-1, or occludin expression (Fig 3). Vehicle-treated control HK-2 monolayers achieved a steady-state TER of 6–8 Ω·cm^2^ when grown on 12-well Transwell filters, compatible with leaky epithelia. Transfection with siRNA against claudin-2 significantly decreased TER compared with controls (4.75 ± 0.32 vs. 7.70 ± 0.48 Ω·cm^2^, *P* < 0.05). On the other hand, TER was significantly increased by transfection with siRNA against occludin (14.03 ± 0.48 Ω·cm^2^, *P* < 0.05) or ZO-1 (11.61 ± 0.18 Ω·cm^2^, *P* < 0.05).

**Fig 4:**
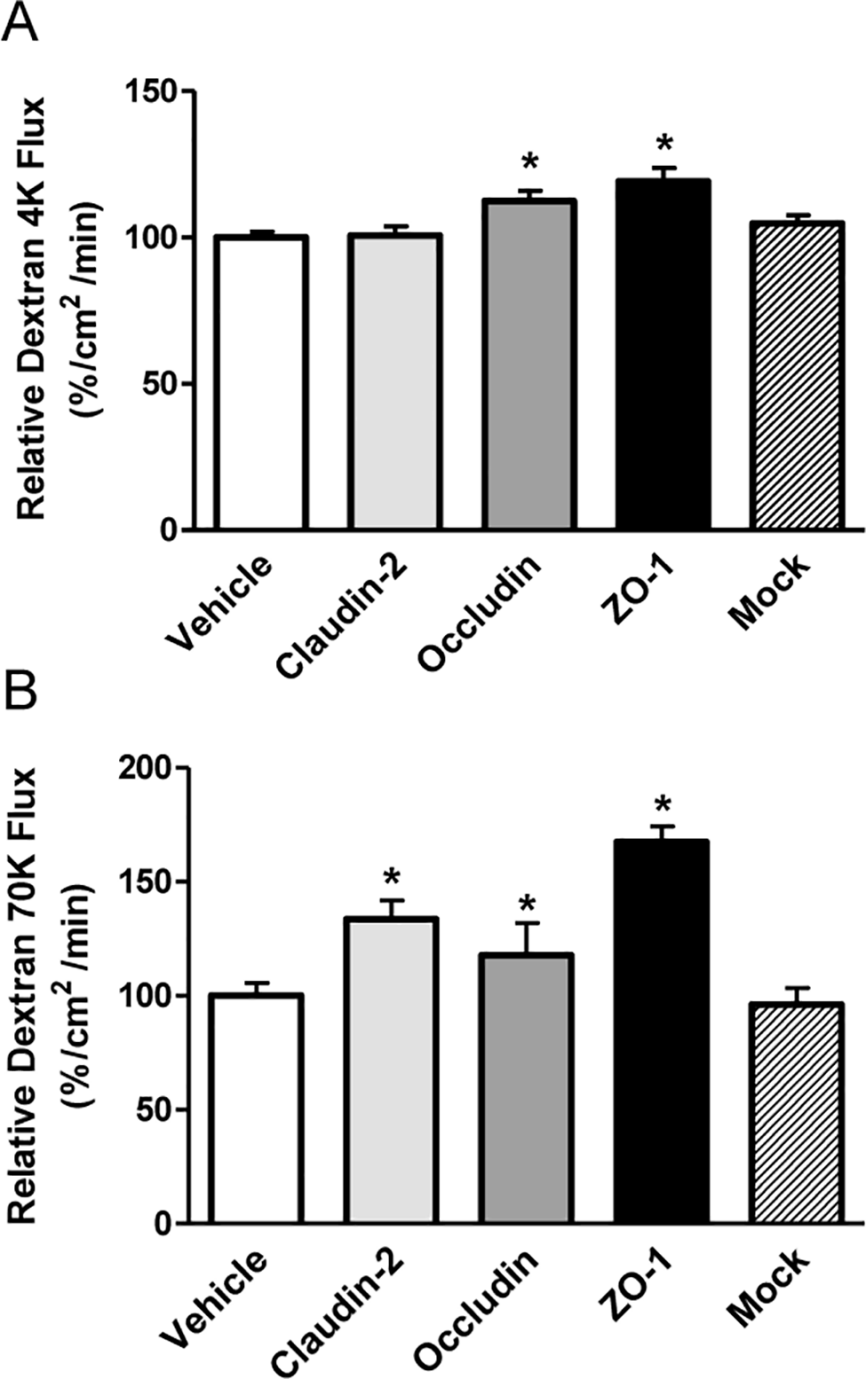
Effects of claudin-2, occludin and ZO-1 siRNA transfection on dextran permeability of HK-2 cell monolayers. A: Flux of 4-kDa FITC-dextran, measured at 24 h, was significantly increased by transfection of siRNA against claudin-2 or occludin. B: Flux of 70-kDa FITC-dextran at 24 h measured epithelial permeability and was significantly increased by siRNA against claudin-2, occludin or ZO-1. Data are mean ± SD. *P < 0.05 by Student’s *t*-test for unpaired data.

To estimate paracellular permeability of HK-2 epithelial monolayers, apical-to-basolateral transepithelial flux rates of FITC-labeled dextran (4 and 70 kDa) were determined. Permeability of HK-2 cells on Transwell inserts to 4-kDa FITC-dextran is presented in Fig 4A. Compared with controls (100 ± 2%), 4-kDa dextran permeability measured at 24 h was significantly increased by siRNA transfection against occludin (113 ± 4%, *P* < 0.05) or ZO-1 (119 ± 5%, *P* < 0.05). However, permeability was not significantly affected by transfection with siRNA against claudin-2 (101 ± 3%). The results of 70-kDa FITC-dextran permeability are in Fig 4B. Compared with controls (100 ± 6%), 70-kDa dextran permeability measured at 24 h was significantly increased by siRNA transfection against claudin-2 (134 ± 8%), occludin (118 ± 14%) or ZO-1 (168 ± 7%).

### Effects of multiple TJ protein knockdown on TER and dextran permeability

**Fig 5:**
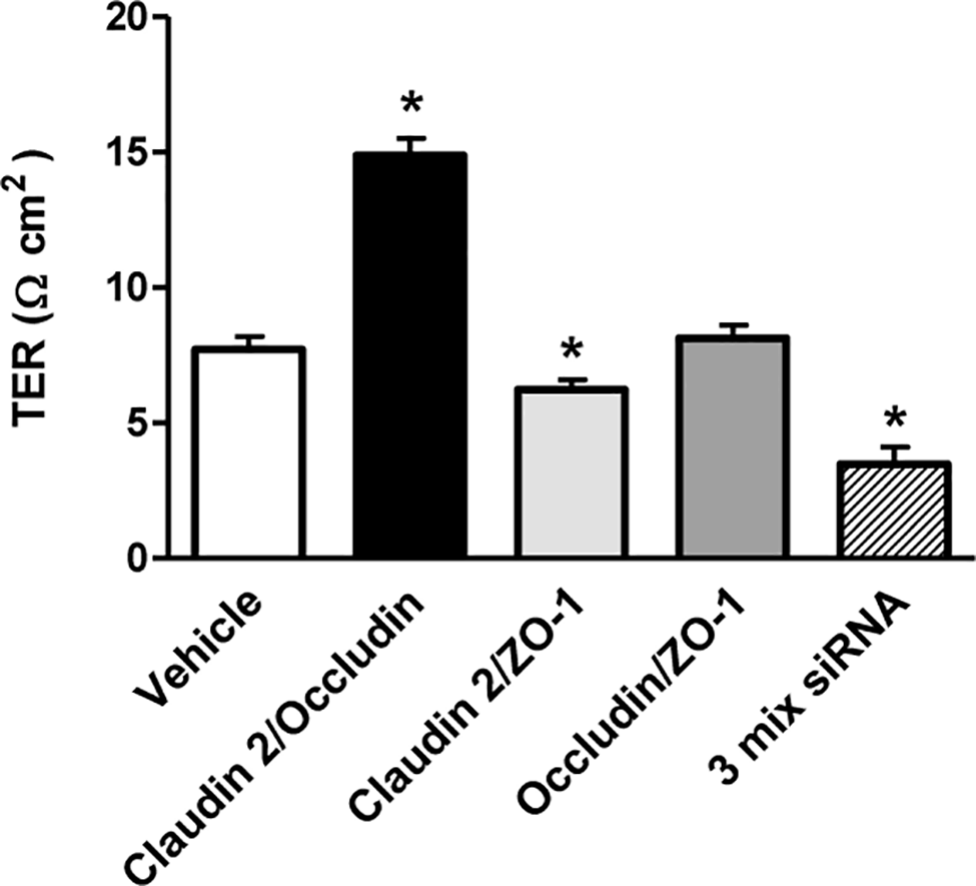
Effects of siRNA-induced multiple knockdown of tight junction proteins on transepithelial electrical resistance (TER) in HK-2 cell monolayers. TER was significantly decreased by claudin-2/ZO-1 siRNA cotransfection, but significantly increased by occluding/claudin-2 siRNA co-transfection. When claudin-2, occludin, and ZO-1 were simultaneously inhibited, TER was suppressed. Data are mean ± SD. *P<0.05 vs. vehicle; by Student’s *t*-test for unpaired data.

We tested whether further changes in TER and dextran permeability were induced by multiple inhibition of claudin-2, ZO-1, or occludin expression in HK-2 cells. Compared with vehicle-treated controls, cotransfection of siRNA against claudin-2 and ZO-1 significantly decreased TER (6.2 ± 0.4 vs. 7.7 ± 0.5 Ω·cm^2^, *P* < 0.05). No significant change in TER was induced by cotransfection of siRNA against ZO-1 and occludin (8.1 ± 0.5 Ω·cm^2^). TER was significantly increased by co-transfection of siRNA against occludin and claudin-2 (14.9 ± 0.6 Ω·cm^2^, *P* < 0.05). When all three TJ proteins were inhibited, TER was suppressed (3.5 ± 0.6 Ω·cm^2^, *P* < 0.05) compared with vehicle-treated controls (Fig 5).

**Fig 6:**
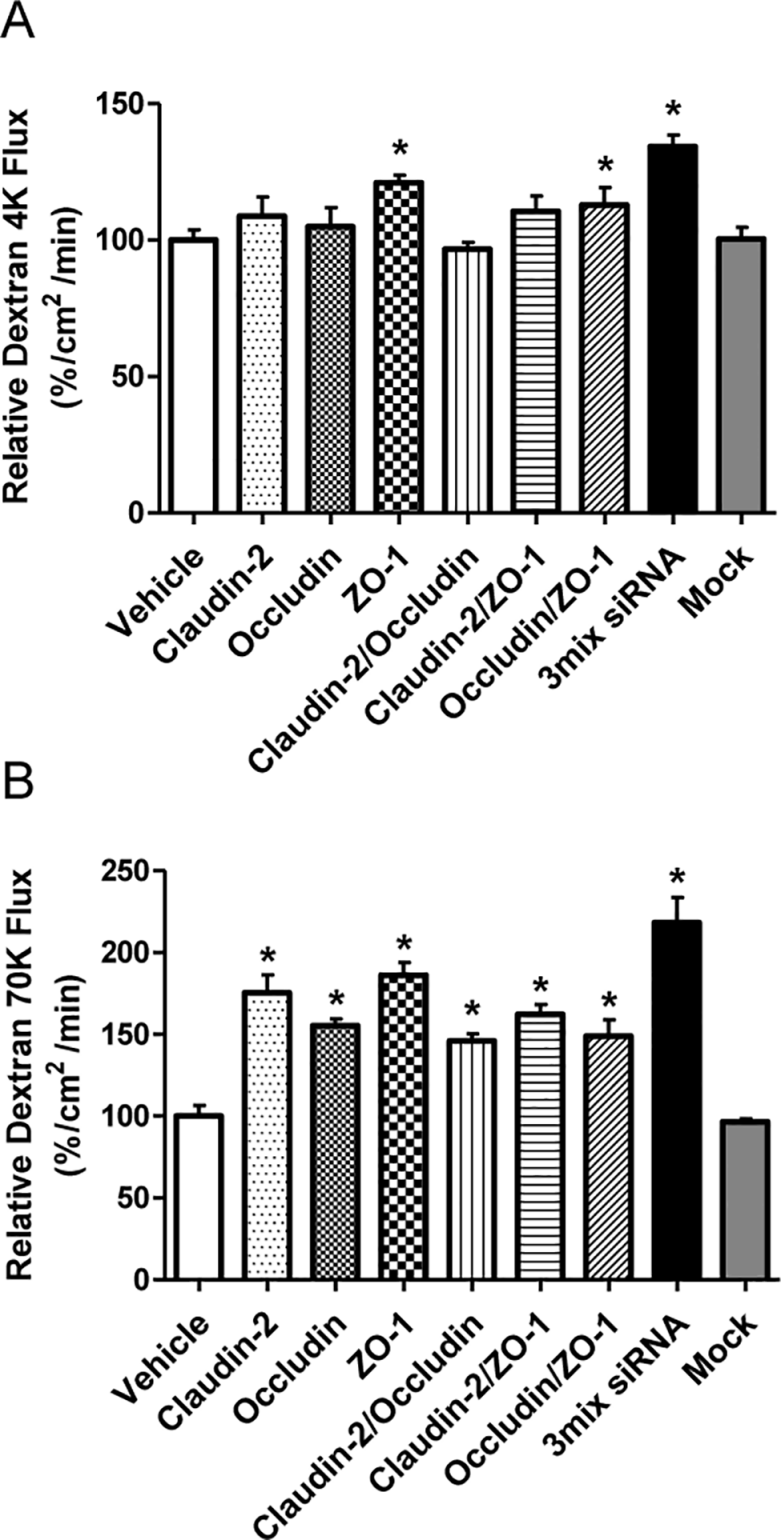
Effects of siRNA-induced multiple knockdown of tight junction proteins on dextran permeability in HK-2 cell monolayers. A: Measurements of 4-kDa FITC-dextran flux at 24 h with significant increases with siRNA cotransfection against occludin and ZO-1 or claudin-2 and occludin/ZO-1. B: Flux at 24 hrs of 70 kDa FITC-dextran flux was used to measure epithelial permeability, and was significantly increased by transfection with all combinations of siRNA. Data are means ± SD. *P< 0.05 vs. vehicle; by Student’s *t*-test for unpaired data.

The effects of multiple inhibitions of TJ proteins on dextran permeability in HK-2 cell monolayers are shown in Fig 6. Compared with controls (100 ± 3.8%), cotransfection of siRNA against occludin and ZO-1 significantly increased 4 kDa FITC-dextran flux (113 ± 6.3%, *P* < 0.05). Flux of 70-kDa FITC-dextran in Fig 6B was consistent with previous single-knockdown experiment (Fig 4B). Thus, 70-kDa FITC-dextran flux was increased by cotransfection of siRNA against claudin-2 and ZO-1 (162 ± 6%, *P* < 0.05), claudin-2 and occludin (146± 4%, *P* < 0.05) or occludin and ZO-1 (149 ± 10%, *P* < 0.05). Flux of 4-kDa (134 ± 4.1%, *P* < 0.05) and 70-kDa (218 ± 15%, *P* < 0.05) FITC-dextran flux increased further when all three TJ proteins were knocked down.

## Discussion

In this study, we demonstrated that claudin-2, occludin, and ZO-1 were strongly expressed and had functional capacity in HK-2 cells, representing the renal proximal tubular epithelium. The siRNA-induced knockdown of claudin-2, occludin or ZO-1 induced changes in TER and paracellular permeability. Furthermore, it accompanied alteration of other adjacent TJ protein expression in HK-2 cells.

We used HK-2 cells to study TJ proteins in the kidney because paracellular transport is important for the iso-osmotic absorption of NaCl in the proximal tubule [14]. In the distal nephron, where transport is predominantly transcellular, tight epithelia may be more important [15]. We previously showed that distal renal TJ proteins are dysregulated in cyclosporine nephrotoxicity [16]. This study suggested different functions for TJ proteins in HK-2 cells. When the claudin-2 gene was depleted, TER decreased. This result was unexpected because TER is lower when claudin-2 is introduced into the MDCK I renal cell line, which lacks claudin-2 [17]. We speculate that endogenous claudin-2 protein in HK-2 cells may have different properties from exogenous claudin-2 overexpression in MDCK I cells. Interestingly, the expression of claudin-2 protein was suppressed by transfection of siRNA against occludin or ZO-1. These interactions suggested that claudin-2 may be directly or indirectly affected by other TJ proteins. The suppression of claudin-2 protein expression was associated with increased 70-kDa FITC-dextran flux, but the direction of change in TER was inconsistent. Our results appear to be compatible with the concept that in the kidney, paracellular permeability is principally determined by claudins that are differentially expressed in each tubule segment [2].

Few previous studies investigated the roles of occludin and ZO-1 in HK-2 cells. Occludin is ubiquitously expressed for barrier formation against macromolecule passage, but its function in the TJ barrier has not yet been elucidated [18]. We showed that TER was enhanced when the occludin gene was inhibited. This finding was unexpected because occludin increases TER in overexpression experiments in MDCK cells [19]. According to Raleigh et al., occludin S408 phosphorylation decreases TER in Caco-2 cells [20]. They concluded that beyond regulating the TJ leak pathway, occludin contributes to control of pore pathway permeability. In contrast, transepithelial permeability measured by dextran flux increased with inhibition of the occludin gene. This discrepancy between paracellular permeability and TER was compatible with results from Balda et al. [21]. Thus, the function of endogenous occludin in HK-2 cells would differ from exogenous occludin in MDCK cells.

We found that expression of occludin increased with transfection of siRNA against claudin-2 into HK-2 cells. Interaction between claudin-2 and occludin might be possible, although the occludin cytoplasmic tail is not reported to interact directly with claudins [20]. TER results after claudin-2 gene depletion (or increased occludin protein expression) are compatible with those from occludin gene inhibition in this study.

ZO-1 is a TJ protein that interacts with both occludin and claudins [22]. In our experiments, ZO-1 gene depletion decreased the expression of claudin-2 protein but did not change the expression of occludin protein. These results were associated with increased TER, suggesting that claudin-2 was critical for transepithelial resistance in HK-2 cells. Among claudin interactions, binding of claudins to ZO-1 is critical for tight junction assembly [23].

We also examined the effects of simultaneous depletion of TJ proteins in HK-2 cells. Our results of multiple inhibition of claudin-2, occludin, and ZO-1 expression were difficult to interpret. TER responded variably to transfection of different combinations of siRNA into HK-2 cells. The 70-kDa FITC-dextran flux increased with all possible combinations of siRNA transfection, however. TER was markedly decreased when all three genes for TJ proteins were inhibited. This change was associated with increased flux of 4-kDa and 70-kDa FITC-dextran, suggesting that integration of claudin-2, ZO-1 and occludin is necessary for maintaining the function of the proximal tubular epithelium.

## Abbreviations

FITC: Fluorescein isothiocyanate
KD: knockdown
siRNA: small-interfering RNAs
TJ: tight junctions
TER: transepithelial electrical resistance
ZO: zonula occludens

## References

[1] Denker BM, Sabath E. The biology of epithelial cell tight junctions in the kidney. J Am Soc Nephrol. 2011;22(4):622–5. doi: 10.1681/ASN.2010090922 21415157 10.1681/ASN.2010090922

[2] Hou J, Rajagopal M, Yu AS. Claudins and the kidney. Annu Rev Physiol. 2013;75:479–501. doi: 10.1146/annurev-physiol-030212-183705 23140368 10.1146/annurev-physiol-030212-183705 PMC3759403

[3] Kirk A, Campbell S, Bass P, Mason J, Collins J. Differential expression of claudin tight junction proteins in the human cortical nephron. Nephrol Dial Transplant. 2010;25(7):2107–19. doi: 10.1093/ndt/gfq006 20124215 10.1093/ndt/gfq006 PMC2891746

[4] Rosenthal R, Gunzel D, Krug SM, Schulzke JD, Fromm M, Yu AS. Claudin-2-mediated cation and water transport share a common pore. Acta physiologica (Oxford, England). 2017;219(2):521–36. doi: 10.1111/apha.12742 27359349 10.1111/apha.12742 PMC5201457

[5] Matter K, Balda MS. Signalling to and from tight junctions. Nat Rev Mol Cell Biol. 2003;4(3):225–36. doi: 10.1038/nrm1055 12612641 10.1038/nrm1055

[6] Shen L. Tight junctions on the move: molecular mechanisms for epithelial barrier regulation. Ann N Y Acad Sci. 2012;1258:9–18. doi: 10.1111/j.1749-6632.2012.06613.x 22731710 10.1111/j.1749-6632.2012.06613.x PMC3690943

[7] Van Itallie CM, Mitic LL, Anderson JM. Claudin-2 forms homodimers and is a component of a high molecular weight protein complex. J Biol Chem. 2011;286(5):3442–50. doi: 10.1074/jbc.M110.195578 21098027 10.1074/jbc.M110.195578 PMC3030350

[8] Al-Sadi R, Khatib K, Guo S, Ye D, Youssef M, Ma T. Occludin regulates macromolecule flux across the intestinal epithelial tight junction barrier. Am J Physiol Gastrointest Liver Physiol. 2011;300(6):G1054–64. doi: 10.1152/ajpgi.00055.2011 21415414 10.1152/ajpgi.00055.2011 PMC3119114

[9] Ryan MJ, Johnson G, Kirk J, Fuerstenberg SM, Zager RA, Torok-Storb B. HK-2: an immortalized proximal tubule epithelial cell line from normal adult human kidney. Kidney international. 1994;45(1):48–57. 8127021 10.1038/ki.1994.6

[10] Hou J, Gomes AS, Paul DL, Goodenough DA. Study of claudin function by RNA interference. J Biol Chem. 2006;281(47):36117–23. doi: 10.1074/jbc.M608853200 17018523 10.1074/jbc.M608853200

[11] Kim S, Choi HJ, Jo CH, Park JS, Kwon TH, Kim GH. Cyclophosphamide-induced vasopressin-independent activation of aquaporin-2 in the rat kidney. Am J Physiol Renal Physiol. 2015;309(5):F474–83. doi: 10.1152/ajprenal.00477.2014 26109089 10.1152/ajprenal.00477.2014

[12] Prozialeck WC, Edwards JR, Lamar PC, Smith CS. Epithelial barrier characteristics and expression of cell adhesion molecules in proximal tubule-derived cell lines commonly used for in vitro toxicity studies. Toxicology in vitro. 2006;20(6):942–53. doi: 10.1016/j.tiv.2005.11.006 16387471 10.1016/j.tiv.2005.11.006

[13] Neunlist M, Toumi F, Oreschkova T, Denis M, Leborgne J, Laboisse CL, et al Human ENS regulates the intestinal epithelial barrier permeability and a tight junction-associated protein ZO-1 via VIPergic pathways. Am J Physiol Gastrointest Liver Physiol. 2003;285(5):G1028–36. doi: 10.1152/ajpgi.00066.2003 12881224 10.1152/ajpgi.00066.2003

[14] Rector FC Jr. Sodium, bicarbonate, and chloride absorption by the proximal tubule. The American journal of physiology. 1983;244(5):F461–71. 6303131 10.1152/ajprenal.1983.244.5.F461

[15] Angelow S, Yu AS. Claudins and paracellular transport: an update. Curr Opin Nephrol Hypertens. 2007;16(5):459–64. doi: 10.1097/MNH.0b013e32820ac97d 17693762 10.1097/MNH.0b013e32820ac97d

[16] Lee CH, Kim S, Kang CM, Kim WY, Kim J, Kim GH. Altered expression of tight junction proteins in cyclosporine nephrotoxicity. Am J Nephrol. 2011;33(1):7–16. doi: 10.1159/000322445 21124021 10.1159/000322445

[17] Furuse M, Furuse K, Sasaki H, Tsukita S. Conversion of zonulae occludentes from tight to leaky strand type by introducing claudin-2 into Madin-Darby canine kidney I cells. The Journal of cell biology. 2001;153(2):263–72. 11309408 10.1083/jcb.153.2.263 PMC2169456

[18] Krug SM, Schulzke JD, Fromm M. Tight junction, selective permeability, and related diseases. Seminars in cell & developmental biology. 2014;36:166–76. doi: 10.1016/j.semcdb.2014.09.002 25220018 10.1016/j.semcdb.2014.09.002

[19] Van Itallie CM, Fanning AS, Holmes J, Anderson JM. Occludin is required for cytokine-induced regulation of tight junction barriers. Journal of cell science. 2010;123(Pt 16):2844–52. doi: 10.1242/jcs.065581 20663912 10.1242/jcs.065581 PMC2915885

[20] Raleigh DR, Boe DM, Yu D, Weber CR, Marchiando AM, Bradford EM, et al Occludin S408 phosphorylation regulates tight junction protein interactions and barrier function. The Journal of cell biology. 2011;193(3):565–82. doi: 10.1083/jcb.201010065 21536752 10.1083/jcb.201010065 PMC3087007

[21] Balda MS, Whitney JA, Flores C, Gonzalez S, Cereijido M, Matter K. Functional dissociation of paracellular permeability and transepithelial electrical resistance and disruption of the apical-basolateral intramembrane diffusion barrier by expression of a mutant tight junction membrane protein. The Journal of cell biology. 1996;134(4):1031–49. 8769425 10.1083/jcb.134.4.1031 PMC2120963

[22] Furuse M, Itoh M, Hirase T, Nagafuchi A, Yonemura S, Tsukita S, et al Direct association of occludin with ZO-1 and its possible involvement in the localization of occludin at tight junctions. J Cell Biol. 1994 12;127(6 Pt 1):1617–26. 7798316 10.1083/jcb.127.6.1617 PMC2120300

[23] Van Itallie CM, Anderson JM. Claudin interactions in and out of the tight junction. Tissue barriers. 2013;1(3):e25247 doi: 10.4161/tisb.25247 24665401 10.4161/tisb.25247 PMC3875638

